# Characterization of a Lytic Bacteriophage vB_EfaS_PHB08 Harboring Endolysin Lys08 against *Enterococcus faecalis* Biofilms

**DOI:** 10.3390/microorganisms8091332

**Published:** 2020-08-31

**Authors:** Dan Yang, Yibao Chen, Erchao Sun, Lin Hua, Zhong Peng, Bin Wu

**Affiliations:** 1 State Key laboratory of Agricultural Microbiology, College of Veterinary Medicine, Huazhong Agricultural University, Wuhan 430070, China; danyang6211@163.com (D.Y.); yibaochen@webmail.hzau.edu.cn (Y.C.); erchaosun@163.com (E.S.); hualin0@webmail.hzau.edu.cn (L.H.); 2 The Cooperative Innovation Center for Sustainable Pig Production, Huazhong Agricultural University, Wuhan 430070, China

**Keywords:** *Enterococcus faecalis*, bacteriophage, bacterial killing, genome analysis, endolysin, biofilms

## Abstract

*Enterococcus faecalis* is an opportunistic pathogen that causes illnesses ranging from urinary tract infections to sepsis in humans and animals. However, the overuse of antibiotics has increased rates of drug resistance among *E. faecalis* isolates. Bacteriophages and their derivatives have recently been identified as good candidates for the treatment of drug-resistant bacterial infections. Here, we isolated a virulent *E. faecalis* phage, PHB08, using the double-layer plate method. The bioactivity of the phage was determined via one-step growth curve testing and bacterial killing assays, and whole-genome sequencing was performed using the Illumina HiSeq platform. In addition, protein expression and antibiofilm assays were performed to investigate the activity of the phage lysin. Results showed that PHB08 has a 55,244-bp linear double-stranded DNA genome encoding 91 putative coding sequences. PHB08 inhibited the growth of host strain EF3964 at 37 °C in tryptic soy broth (TSB) medium, while in vegetable models, PHB08 caused a 4.69-log decrease in viable *E. faecalis* cells after 24 h. Both PHB08 and its endolysin lys08 showed antibiofilm activity against *E. faecalis* biofilms, which was enhanced by Mn^2+^ ions. Thus, virulent phage PHB08 and endolysin lys08 may be good candidates for reducing and/or eradicating *E. faecalis* infections.

## 1. Introduction

The Gram-positive bacterium *Enterococcus faecalis* is an important foodborne pathogen [1] capable of causing endocarditis, sepsis, and meningitis in both animals and humans [2,3,4]. Reports suggest that *E. faecalis* is the third-most common cause of hospital-acquired bacterial infection, accounting for 15% of catheter-associated urinary tract infections and 5%–15% of infective endocarditis cases [5,6,7]. *E. faecalis* strains also play a role in periodontal disease and are closely associated with hard-to-treat persistent intra-articular infections [8,9]. In general, *E. faecalis* infections are treated with antimicrobial agents such as vancomycin [10,11,12] or aminoglycosides [13]. However, overuse of these drugs has resulted in the decreased susceptibility of clinical isolates to antibiotics, a worrying trend that has been observed in many bacterial pathogens (https://www.who.int/emergencies/ten-threats-to-global-health-in-2019) [14,15,16]. In addition, antibiotic resistance genes can be transferred among the different *E. faecalis* genotypes, further complicating the clinical treatment of infections [17].

During infection, *E. faecalis* often forms a single- or mixed-species biofilm on host tissues or in-dwelling medical devices, usually when exposed to fluid flow [18,19]. Bacteria contained within a biofilm are protected from the effects of antibiotics, resulting in infections that are recalcitrant to treatment [20]. Therefore, eradication of *E. faecalis* biofilms are of significant clinical benefit.

Phages are natural predators of bacteria [21] and have been investigated as potential therapeutic tools since their discovery in 1915 [22]. Recently, phages have been used as biological control agents to specifically inhibit the reproduction and survival of *E. faecalis* [23,24,25]. For example, an *E. faecalis* bacteriophage was used to control infection following root canal treatment, while another species-specific phage was successfully used to treat a patient with severe septic peritonitis caused by vancomycin-resistant *E. faecalis*. Phages and their derivatives, such as endolysins, are also effective for reducing and/or eradicating bacterial biofilms [26,27,28]. However, few studies have examined the efficacy of phages and/or phage-derivatives in controlling biofilms formed by Gram-positive bacteria, including *E. faecalis*, in vitro [29,30].

In this study, we isolated a phage, designated vB_EfaS_PHB08 (hereafter referred to as PHB08), from hospital sewage using *E. faecalis* strain EF3964 as an indicator. Phage assays confirmed that PHB08 effectively inhibited the growth of *E. faecalis* in liquid medium and on the surfaces of fruit, while both PHB08 and its endolysin displayed potential antibiofilm activity against mature *E. faecalis* biofilms.

## 2. Materials and Methods

### 2.1. Bacterial Strains and Cultural Conditions

*E. faecalis* strain EF3964 was isolated from a patient with urinary tract infections. Eighteen *E. faecalis* strains, ten *E. faecium* strains, four *E. coli* strains, and three *Salmonella* strains were used to test the host range (Table S1). All strains included in this study were grown on tryptic soy agars (TSA; Becton, Dickinson and company, MD, USA) and/or in tryptic soy broth (TSB; Becton, Dickinson and company, MD, USA) at 37 °C for 16 h.

### 2.2. Phage Isolation and Purification

*E. faecalis* phage was isolated from sewage collected from nearby hospitals by using a double-layer plate methodology, as described previously [31]. Briefly, sewage samples were centrifuged at 2100× *g* for 10 min. The supernatants were filtered using a 0.22 μm membrane (Millex–GP, USA) to remove bacteria. After that, 300 μL of the filtrates mixed with 300 μL of the indicator bacterium in exponential growth phase was poured on the double-layer plate, and then incubated at 37 °C for 12 h. A single plaque was picked and then was resuspended in sterile SM buffer (100 mM NaCl, 8.5 mM MgSO_4_·7H2O, 50 mM Tris-Cl (pH 7.5), and 0.01% gelatin) [32]. Thereafter, four rounds of phage purification assays were performed by using the double-layer ager method as described previously [31]. The isolated phages were finally purified through CsCl gradient ultra-centrifugation and then stored at 4 °C [33]. The morphology of the phage was observed under a transmission electron microscope (HITACHI H-7650, Tokyo, Japan).

### 2.3. Host Range of the Phage

The host range of phage PHB08 was evaluated by using the spot test method [34]. A total of 300 μL of the indicator bacterium (including nineteen *E. faecalis* strains, ten *E. faecium* strains, four *E. coli* strains, and three *Salmonella* strains) in the exponential growth phase mixed with 6 mL of soft agar was poured on the double-layer plate. A total of 10 μL of the different titers of the purified phage PHB08 with the different titers (PFU = 10^8^, PFU = 10^5^, and PFU = 10^3^, respectively) were put on a double-layer plate. The plates were incubated at 37 °C for 12 h.

### 2.4. Physical Parameter of the Phage

To test the temperature sensitivity, 100 μL of the purified phages (1 × 10^9^ plaque forming units, PFU) was treated under different temperatures (4, 20, 40, 50, 60, 70, and 80 °C) for 20, 40 and 60 min, respectively. For the acid-base sensitivity assay, 100 μL of phage (1 × 10^9^ PFU) with 900 μL different pH (3.0, 5.0, 7.0, 9.0, and 11.0) SM buffer was treated at 37 °C for 1 h. Phage titers were determined using the double-layer plate method, as described previously [34]. Each of the experiments were repeated three times.

### 2.5. One-Step Growth Curve

One-step growth curve of phage isolated was determined as described previously [31]. Briefly, the phage with multiplicity of infection (MOI) = 0.01 was inoculated into 20 mL of the indicator bacteria in exponential growth phase. The mixture was incubated at 37 °C for 5 min. After incubation, the mixture was centrifuged at 12,000× *g* for 30 s. The pellets were resuspended with equal volume TSB medium, and oscillatory culture (37 °C) was started. Phage titer was measured every 10 min for 130 min. The titer of phage was determined by double-layer plate method, as described previously [34]. The latent period of every phage was calculated as the time interval between adsorption and the beginning of the first burst. The burst size was calculated as the ratio of the final count of released phage progeny to the initial count of infected bacterial host cell during the latent period using the following formula: The burst size (PFUs) = (final titer (PFUs) − initial titer (PFUs))/initial titer (PFUs) [35]. This experiment was repeated at least three times.

### 2.6. Lytic Activity

The lytic activity of the phage was analyzed in a 96-well microtiter plate by examining the optical density (OD_600_) measurement method [36,37]. Briefly, 100 μL of the *E. faecalis* EF3964 (6.6 × 10^7^ colony forming units, CFU) in exponential growth phase mixed with 100 μL of phage at different MOIs (0.0001, 0.001, 0.01, 0.1, 1.0, 10, 100, and 100) were shaken at 37 °C with 180 rpm. Wells with an equal volume of TSB medium or phosphate buffer saline (PBS, PH = 7.5) buffer added were used as controls. OD_600_ values were determined by a multimode microplate reader (Tecan Spark 10M, Switzerland). This experiment was performed in triplicate.

The effect of phage PHB08 on killing *E. faecalis* EF3964 was evaluated in vegetable (lettuce) models, as previously described [38]. Briefly, the vegetable was sterilized with sodium hypochlorite (100 μg/mL) for 5 min. After washing with sterile water, vegetables were covered evenly with host strain EF3964 (10^5^ CFU/cm^2^) until the sample dried naturally. Subsequently, the phage with different MOI values (1000, 100, 10, and 1.0) was sprayed on the vegetables, which were then incubated at 25 °C or 4 °C for 6, 12, and 24 h, respectively. The control group was treated with the equal volume PBS. Bacteria were resuscitated from the vegetables and were counted by 10-fold dilutions method. This experiment was performed in triplicate.

### 2.7. DNA Extraction and Analysis of Genome Sequence

The phages’ genomic DNA was extracted by using the phenol-chloroform protocol [39] and was then sequenced on an Illumina HiSeq 2,500 platform with a 2 × 100 bp read length. The short reads were assembled by SOAPdenovo [40]. Open reading frames (ORFs) were predicted using Glimmer [41,42]. The final assembled sequence was searched against the current protein and nucleotide databases (http://www.ncbi.nlm.nih.gov/) by means of the basic local alignment search tool (BLAST). The genomes were scanned for tRNAs using tRNA scan-SE [43] and ARAGORN [44]. Protein BLAST (BLASTP) was used to identify putative homologies and proteins sharing similarities with the predicted phage proteins. Gene analysis of phage resistance was performed by ResFinder (https://cge.cbs.dtu.dk/services/ResFinder/). The phylogenetic tree was analyzed using the ClustalW program in MEGA X [45].

### 2.8. Cloning, Expression and Activity Identification of the Endolysin

The putative endolysin encoding gene of the phage PHB08 was amplified by polymerase chain reaction (PCR) assays with specific primers (5′-CGTGTGTCACATACCTGAATTG-3′; 5′-GCAGTAACAGCCATTCATCTATG-3′), which was then cloned into the expression vector pET-28a(+) (EMD Biosciences, USA), generating pET-lys08, which was finally transformed into *E.coli* BL21 (DE3). Protein expression was induced by 0.6 mM/L IPTG at 25 °C for 16 h. Ni-nitrilotriacetic acid columns were used to purify the protein expressed, as previously described [46]. Purified protein was filtered using a 0.45 μm membrane and then was concentrated by a 3-kDa ultrafiltration tube (Solarbio, Beijing, China). Purified protein was quantified by the Bradford Protein Assay Kit (Thermo Fisher Scientific, USA) and stored at −80 °C until use.

### 2.9. Antibiofilm by Phage and Its Endolysins

Biofilm formation was detected using a 96-well microtiter plate as previously described [31,47,48]. Briefly, the overnight cultured bacterial strain was diluted in 1:100 solution. A total of 100 μL of the diluent bacteria was added to each well and equal volumes of medium were included as control. These plates were incubated at 37 °C for 24 and 48 h. Each well was washed three times with sterile PBS buffer. In this process, two parallel tests were carried out to measure the absorbance value of resulting supernatant and the number of active biofilm cells (CFU/cm^2^), respectively. For the absorbance value of resulting supernatant assay, 100 μL of the phage PHB08 isolated (1.0 × 10^8^ PFU/mL and 1.0 × 10^7^ PFU/mL) or 100 μL of the endolysin (50 μg and 100 μg) was added to every well and then incubated at 37 °C for 4 h. After incubation, the liquid in each well was discarded, 200 μL of methanol was used to fix the cells for 30 min, dried naturally and added 200 μL of 1% crystal violet was added for 15 min. Each plate was washed three times with sterile PBS buffer and then 150 μL of 33% acetic acid was added to each well. The absorbance value of resulting supernatant was measured at OD_590_ as mentioned above. For the number of active biofilm cells assay, the biofilm matrix of each well was resuspended with sterile PBS buffer. The number of active biofilm cells (CFU/cm^2^) was calculated by 10-fold serial dilution. The experiment was performed in three times.

The effect of different metal ions mixed endolysin proteins on biofilm was evaluated as previous described, with minor modification [49,50]. A 100 μL of different metal ions Mn^2+^ (0.1 mM, 1.0 mM, and 10 mM final concentration, respectively) and Mg^2+^ (0.1 mM, 1.0 mM, and 10 mM final concentration, respectively) was added to endolysin lys08 (50 μg) as above described. The effect of different metal ions mixed with endolysin proteins on biofilm was evaluated as previously described, with minor modification [49,50]. A total of 100 μL of endolysin lys08 (50 μg) was added different metal ions Mn^2+^ (0.1, 1.0, and 10 mM final concentration, respectively) and Mg^2+^ (0.1, 1.0, and 10 mM final concentration, respectively) as above described.

## 3. Results

### 3.1. Characteristics of Phage Specific for E. faecalis

**Figure 1:**
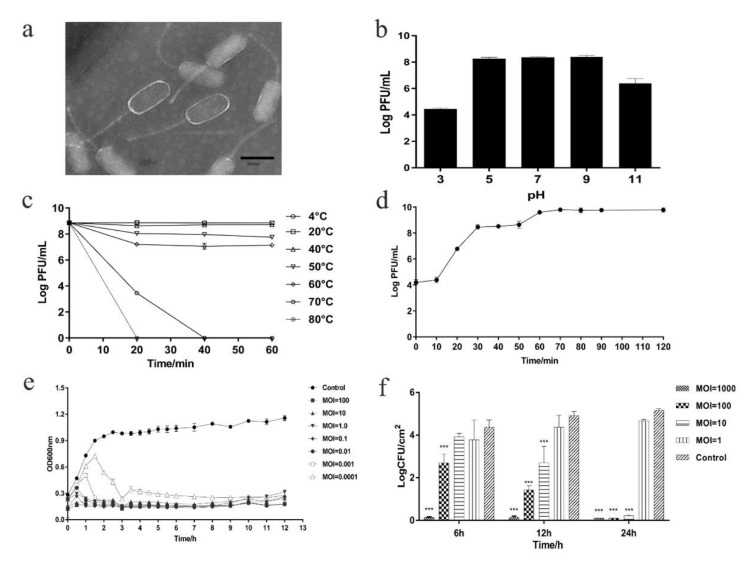
The characteristics of phage PHB08. (**a**) Electron microscopy of phage PHB08. Phage PHB08 has a rectangular head (length 124 mm ± 5, width 61 mm ± 5) and a long tail (158 mm ± 5). The scale in the right corner is 200 nm. (**b**) Stability of phage PHB08 at different pH values. (**c**) Stability of phage PHB08 at different temperatures. (**d**) Curves for one-step growth of phage PHB08. (**e**) Killing assay in tryptic soy broth (TSB) medium. (**f**) Killing assay in vegetable module. Data are expressed as the means ± SDs. Significance was determined by analysis of variance (ANOVA) (*** *p* < 0.001).

The isolated *E. faecalis* phage, designated PHB08, formed a clear, translucent, uniform size plaque on a double-layer agar plate. Under an electron microscope, PHB08 had a rectangular head (length 124 mm ± 5, width 61 mm ± 5) and a long tail (158 mm ± 5) (Figure 1a). Based on these morphological characteristics and according to the latest International Committee on Taxonomy of Viruses (ICTV) classification, PHB08 was determined as a member of the family *Siphoviridae*.

Acid-base tolerance results revealed that the activity of PHB08 was stable between pH 5.0–11.0 (Figure 1b). Temperature tolerance results showed that the titer of phage PHB08 was stable between 4 to 60 °C (Figure 1c). One-step growth curve revealed that PHB08 had a latency of 10 min and a high-speed growth period of 50 min with an average burst size of 64 phage particles per infected cell after 70 min at 37 °C (Figure 1d). Host range assays indicated that phage PHB08 specifically infected 15 out of 19 *E. faecalis* clinical isolates tested, but did not infect other bacterial species including *Enterococcus faecium* (Table S1).

The effects of phage PHB08 on *E. faecalis* host strain EF3964 were evaluated over a 12-h experimental period in liquid medium at 37 °C. As shown in Figure 1e, phage PHB08 showed strong antibacterial activity at a range of MOI values (MOI = 0.0001–100). From 2 h post inoculation, growth curves based on OD_600_ values showed a decreasing trend, indicating that the host bacteria were killed by phage PHB08. Lytic activity tests using vegetable models revealed that PHB08 also killed host bacterial cells on the surfaces of vegetable at 25 °C. PHB08 treatment at MOI values of 1000 and 100 resulted in approximately 4.69-log and 3.41-log reductions in viable *E. faecalis* EF3964 cells, respectively, compared with the control. However, no significant decrease in host cell viability was observed over the 24-h experimental period following PHB08 treatment at MOI = 1 (Figure 1f) or when assays were conducted at 4 °C.

### 3.2. General Feature of Phage PHB08 Whole Genome Sequence

**Figure 2:**
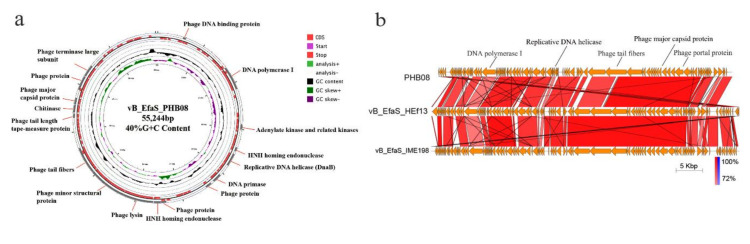
(**a**) Circular genetic map of phage PHB08. The red part represents the distribution of the CDS region. The black part represents the total content of GC (40%). The green part represents the GC skew^+^, which means the GC shift on the leading chain is positive and the purple is the GC skew^−^. (**b**) Homology analysis of phage PHB08 with vB_EfaS_HEf13 and vB_EfaS_IME198.

**Figure 3:**
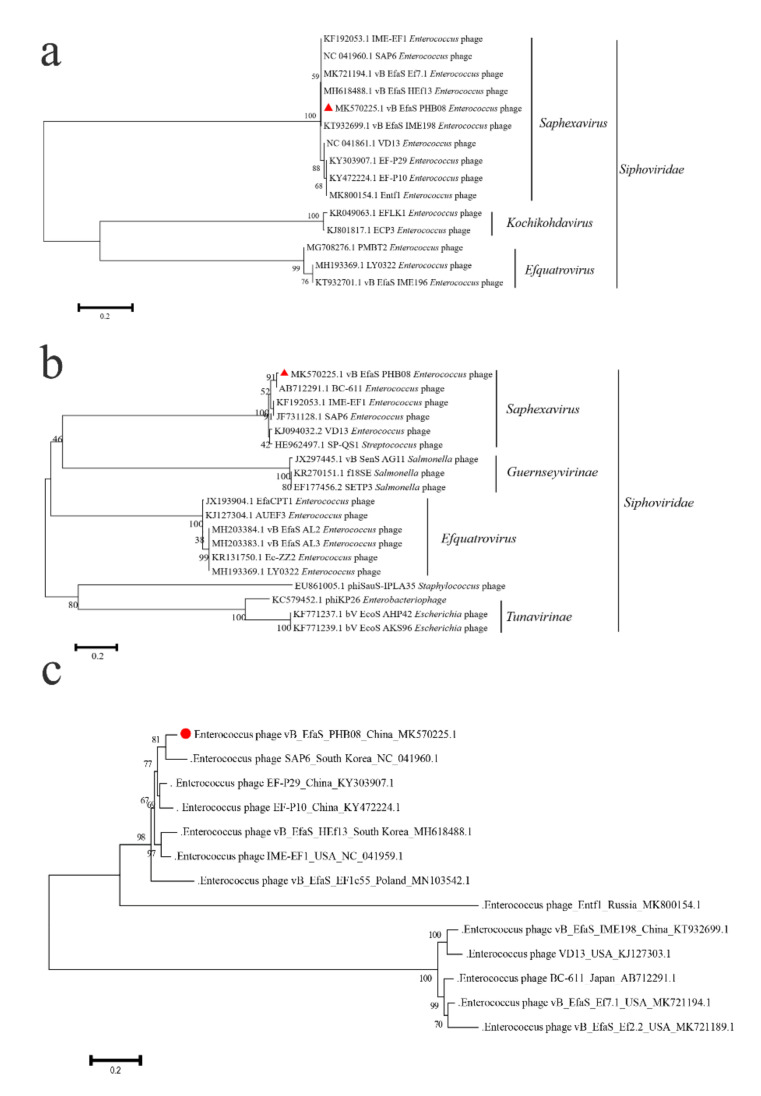
Cluster analysis of phage PHB08. (**a**) The amino acid of terminase large subunit and the major capsid proteins (**b**) of phage PHB08 was analyzed by MEGA X. (**c**) Cluster analysis of *Enterococcus faecalis* phages from different countries. The neighbor-joining method was used to construct phylogenetic with a bootstrap re-sampling analysis of 1000 replicates. The numbers next to the branches are bootstrap values and the red symbols (a,b: red triangle, c: red solid dot) represents the branch of phage PHB08.

Whole genome sequencing revealed that PHB08 had a linear double-stranded DNA genome of 55,244-bp in length, with a G+C content of 40.06% (Figure 2a). The general features of the PHB08 genome sequence were similar to those of the other published *E. faecalis* phage genomes (Table S2). Sequence alignments revealed that the genome sequence PHB08 shared 96.18% and 95.95% DNA identities to those of phage vB_EfaS_IME198 (GenBank accession no. KT932699.1) and vB_EfaS_HEf13 (GenBank accession no. MH618488.1), respectively (Figure 2b). At total of 91 predicted proteins and one tRNA (Trp-CCA) were encoded by the genome sequence of PHB08. Proteins involved in structure and assembly were encoded protein (e.g., tail fiber (CDS61), tail length tape-measure protein (CDS62), major capsid protein (CDS71), portal protein (CDS74), and terminase large subunit (CDS75). Genes encoding DNA replication and regulation modules such as (e.g., DNA binding protein (CDS6), DNA polymerase I (CDS22), adenylate kinase and related kinases (CDS35), HNH homing endonuclease (CDS39, CDS58, and CDS87), replicative DNA helicase (CDS41), DNA replication protein (CDS42), and DNA primase (CDS44) (Table S3). In particularly, a putative endolysin protein (CDS59) was predicted. This protein showed 96% amino acid similarity to the CHAP domain protein of *Enterococcus* phage Entf1 (GenBank accession no. MK800154.1) (Figure S1). The alignment of the endolysin of phage PHB08 and the other 10 strains of *E. faecalis* phages showed that the two major mutations of the amino acid in PHB08 endolysin were observed (Pro→Ala at position 138 and Lys→Ala at position 147) (Figure S1). No lysogeny-associated gene and genes encoding known antibiotic resistance were predicted. Phylogenetic analysis indicated that PHB08 belonged to genus *Saphexavirus*, family *Siphoviridae* (Figure 3a–c).

### 3.3. Antibiofilm Activity

**Figure 4:**
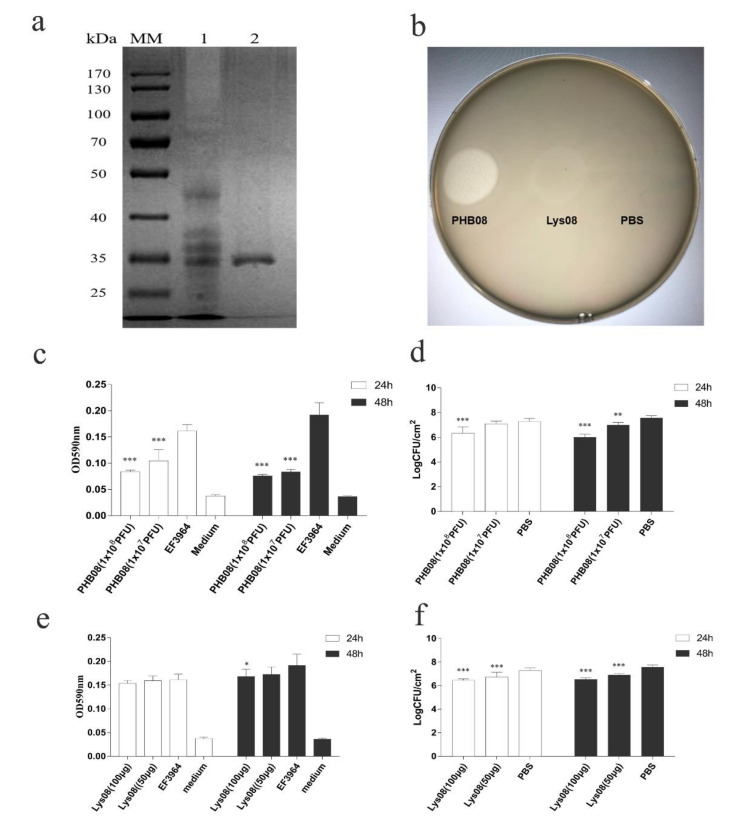
Antibiofilm activity. (**a**) Sodium dodecyl sulfate (SDS) polyacrylamide gel electrophoresis analysis of purified protein lys08. Lane 1: Unpurified protein, lane 2: Purified protein. (**b**) The activity of PHB08 and purified lys08 was determined by spot tests. A total of 5 μL of endolysin lys08 (5 μg) was spotted on the plated containing host strain EF3964 at 37 °C for 12 h. (**c**) The value of OD_590_ after phage PHB08 with different titers was applied to biofilm. (**d**) Determination of the number of living cells with phage PHB08 treated. (**e**) Values of OD_590_ at different concentrations of lys08 on biofilm. (**f**) Determination of the number of living cells with enzyme lys08 treated. The bacteria were counted by 10-fold dilutions method. Three independent experiments were performed, and data are expressed as means ± SD (*n* = 3). Significance was determined by ANOVA ((* *p* < 0.05, ** *p* < 0.01 and *** *p* < 0.001).

**Figure 5:**
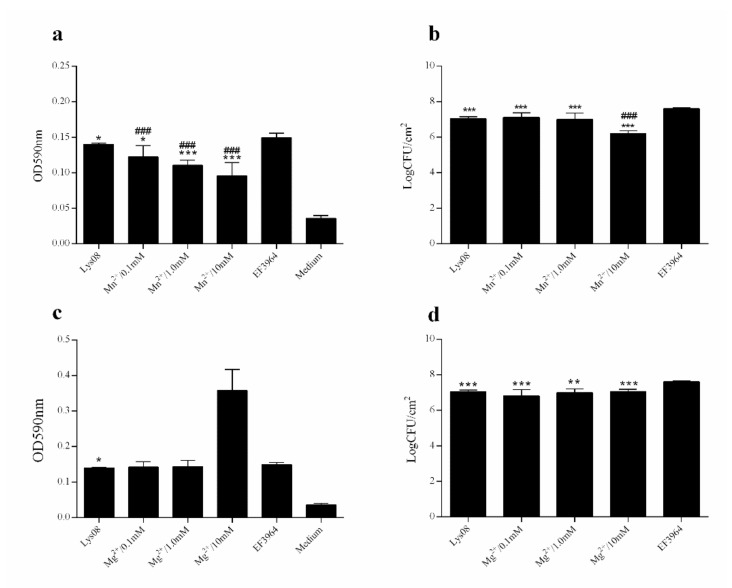
Effects of divalent ions on the activity of endolysin lys08. (**a**,**c**) OD_590_ values of wells following treatment of *E. faecalis* EF3964 biofilms with endolysin lys08 plus various concentrations of Mn^2+^ or Mg^2+^ ions. (**b**,**d**) Viable cell counts following treatment of *E. faecalis* EF3964 with endolysin lys08 plus various concentrations of Mn^2+^ or Mg^2+^ ions. Significant differences between the lys08-treated and untreated (EF3964) groups were determined by ANOVA (* *p* < 0.05, ** *p* < 0.01, and *** *p* < 0.001). For the comparisons between the lys08 plus ions treatment groups and the lys08-treated groups, significance is indicated by ### *p* < 0.001. Data are expressed as means ± SD from three independent experimental replicates (*n* = 3).

To assess the antibiofilm activity of PHB08 and its endolysin, the predicted endolysin protein (CDS59) was expressed in *E. coli* BL21 (DE3). The strategy yielded an approximately 26.4-kDa protein following expression and purification (Figure 4a). Spot tests revealed that both host phage PHB08 and the expressed protein lysed *E. faecalis* strain EF3964 (Figure 4b). Further, antibiofilm tests showed that both phage PHB08 and its endolysin, lys08, significantly reduced *E. faecalis* biofilm density (Figure 4c–f). In co-culture for 4 h at 37 °C, PHB08 significantly diminished biofilm formation (*p* < 0.001) (Figure 4c,d), with a significant difference also observed between the lys08-treated and control groups (*p* < 0.001) (Figure 4e,f). The effects of Mg^2+^ and Mn^2+^ on the lytic activity of the endolysin were then examined. A 0.82-log reduction in viable host cell counts was observed following treatment with lys08 plus Mn^2+^(10 mM) compared with the lys08-treated group (Figure 5a,b). No significant difference was observed between the lys08 plus Mg^2+^ group and the lys08-treated group (Figure 5c,d).

## 4. Discussion

Recently, phages and their derivates have been re-considered as effective tools for treating bacterial infections, particularly those caused by multi-drug-resistant pathogens [30,51,52]. The emergence of resistance in *Enterococci* against antibiotics of glycopeptides (vancomycin and teicoplanin) has attracted people’s attention [53,54]. In this study, we isolated a virulent *E. faecalis* phage PHB08 from sewages nearby to the hospital. Morphological characterization revealed that PHB08 had a rectangular head (length 124 mm ± 5, width 61 mm ± 5) and a long tail (158 mm ± 5) (Figure 1a). These morphological characteristics were similar to *Enterococcus* phage HEf13 [55] and phage IME-EF1 [56]. The phylogenetic analysis showed that PHB08 was closely related to phage SAP6 (isolated from South Korea) (Figure 3c). The results indicated those phages, which were isolated from different countries (China, USA, Japan, South Korea, Poland, Russia) may have evolved from the same ancestor.

Characterizations on the temperature and pH stabilities showed that PHB08 had relatively excellent stability at temperatures between 4 °C and 60 °C, and pH between 5.0 and 9.0, which display a similar characteristics to those of the other *E. faecalis* phages, such as *Enterococcus* phage vB_EfaS_HEf13 [55], and *Enterococcus* phage EF-P10 [57]. One-step growth curve revealed that PHB08 had a latency of 10 min and a high-speed growth period of 50 min with an average burst size of 64 phage particles per infected cell after 70 min at 37 °C (Figure 1d), which is in agreement with the generally reported average burst of phages (every infected cell contained about 30–122 phage particles) [58,59]. Killing assays showed that PHB08 was able to lyse its host strain at different MOI for 12 h in medium (Figure 1e). Potential bactericidal ability at low MOI was similar to those of the single phage 13076 and phage 14028 [36]. Characterizations on the temperature and pH stabilities showed that PHB08 had relatively excellent stability at temperatures between 4 and 60 °C, and pH between 5.0 and 9.0, which displays similar characteristics to those of the other *E. faecalis* phages, such as *Enterococcus* phage vB_EfaS_HEf13 [55], and *Enterococcus* phage EF-P10 [57]. One-step growth curve revealed that PHB08 had a latency of 10 min and a high-speed growth period of 50 min with an average burst size of 64 phage particles per infected cell after 70 min at 37 °C (Figure 1d), which is in agreement with the generally reported average burst of phages (about 30-122 phage particles per infected) [58,59]. Killing assays showed that PHB08 was able to lyse its host strain at different MOIs for 12 h in medium (Figure 1e). Potential bactericidal ability at a low MOI was similar to those of the single phage 13076 and phage 14028 [36].

*E. faecalis* is commonly isolated from the surfaces of foods such as lettuce, spinach, and raw chicken [60,61,62]. In the current study, treatment of *E. faecalis* strain EF3964 with novel phage PHB08 resulted in a significant decrease (4.69-log reduction) in viable cell numbers compared with the control at 25 °C (Figure 1f). However, at lower temperatures, a higher MOI was required to kill or inhibit host bacteria. At 4 °C, no significant difference in the number of viable bacteria was observed between the phage-treated and control groups. This phenomenon may be representative of abortive infection and/or lysis, events that are usually associated with T-even and other large-genome phages, but which are not particularly widespread [63]. A higher MOI of PHB08 may be needed to further verify this hypothesis.

Phages and their derivatives can degrade the outer layer of host cells, thus destroying the integrity of a biofilm [31]. In our study, no significant difference in OD_590_ values was observed between control- and lys08-treated 24-h-old biofilms (Figure 4e). However, a 0.83-log reduction in the number of viable cells was observed in the lys08-treated biofilm compared with the control (Figure 4f). One possible explanation is that crystal violet staining reflects the total biomass in a biofilm, including both living and dead cells, polysaccharides, and nucleic acids [31,36,64]. Because endolysins are single proteins, they are likely to be more suitable than entire phages as biological agents. We therefore studied the effects of ions on the activity of endolysin lys08. A significant difference in OD_590_ values was observed between the lys08 plus Mn^2+^ (10 mM final concentration)-treated and lys08-only-treated groups (Figure 5a), which was reflected in the 0.82-log reduction in live bacterial cell counts in the lys08 plus Mn^2+^-treated group compared with the lys08-treated group (*p* < 0.001) (Figure 5b) [49,50,65].

In conclusion, we isolated a virulent phage PHB08 specific for *E. faecalis*. Our results suggested PHB08 and its endolysin lys08 as potential biocontrol agents could diminish the biofilm formation of *E. faecalis*.

## Supplementary Materials

The following are available online at https://www.mdpi.com/2076-2607/8/9/1332/s1, Figure S1: Phylogenetic analysis and the alignment of the endolysin of phage PHB08 and other 10 strains of *E. faecalis* phages. Table S1: The host range of PHB08. Table S2: Sequence information for the *E. faecalis* phages belonging to *Saphexavirus* subfamily used in this study. Table S3: Gene annotation for each CDS of PHB08.
