# Supplementary material for: Characterization of a Lytic Bacteriophage vB_EfaS_PHB08 Harboring Endolysin Lys08 against Enterococcus faecalis Biofilms
Source: Microorganisms. 2020 Aug 31;8(9):1332. doi: 10.3390/microorganisms8091332 (PMC7564645; doi:10.3390/microorganisms8091332)
Supplement: Supplementary file 1 [file microorganisms-08-01332-s001.pdf]

# Characterization of a Lytic Bacteriophage vB_EfaS_PHB08 Harboring Endolysin Lys08 Against *Enterococcus faecalis* Biofilm

Dan Yang, Yibao Chen, Erchao Sun, Lin Hua, Zhong Peng, Bin Wu

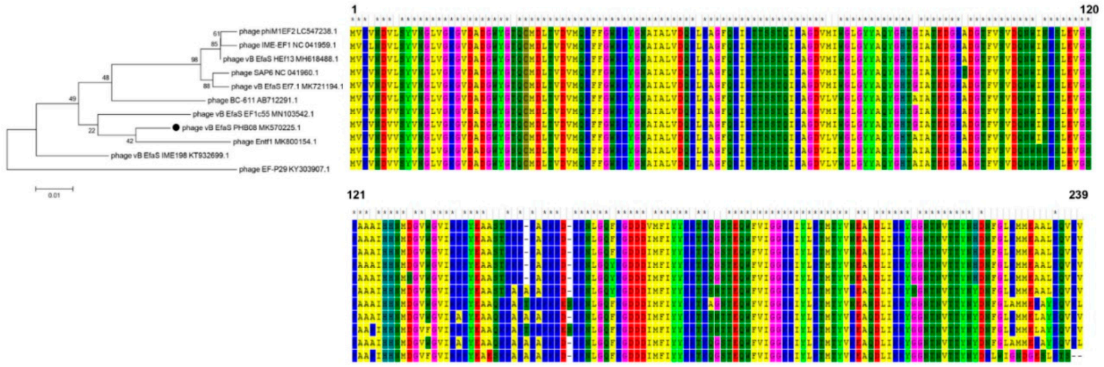


**Figure S1. Phylogenetic analysis and amino acid sequence alignment of the endolysin of phage PHB08 and other 10 strains of *E. faecalis* phages.** Phylogenetic tree was analyzed with the maximum likelihood algorithm and bootstrap values were calculated with 500 replicates by MEGA X. The numbers next to the branches are bootstrap values. The alignment of the endolysin of phage PHB08 and other 10 strains of *E. faecalis* phages showed that the two major mutations of the amino acid in PHB08 endolysin was observed (Pro→Ala at position 138 and Lys→Ala at position 147)

## Tables

**Table S1 The host range of PHB08.**

| Strains | Isolated locations | Phage PHB08 PFU=$10^8$ | Phage PHB08 PFU=$10^5$ | Phage PHB08 PFU=$10^3$ |
|---|---|---|---|---|
| *E. faecalis* EF3964 (Host Strain) | Hubei, China | + | + | + |
| *E. faecalis* EF1833 | Hubei, China | + | + | + |
| *E. faecalis* EFHB01 | Hubei, China | + | + | + |
| *E. faecalis* EFHB02 | Hubei, China | + | + | + |
| *E. faecalis* EFHB03 | Hubei, China | + | + | + |
| *E. faecalis* EFHB04 | Hubei, China | - | - | - |
| *E. faecalis* EF1067 | Anhui, China | + | + | + |
| *E. faecalis* EF7011 | Anhui, China | + | + | + |
| *E. faecalis* EFhn15 | Hunan, China | + | + | + |
| *E. faecalis* EFhn20 | Hunan, China | - | - | - |
| *E. faecalis* FJ1801 | Fujian, China | + | + | + |
| *E. faecalis* Hu6 | Unknow | + | + | + |
| *E. faecalis* Hu7 | Unknow | + | + | + |
| *E. faecalis* Hu8 | Unknow | - | - | - |
| *E. faecalis* XN01 | Shanxi, China | + | + | + |
| *E. faecalis* XN02 | Shanxi, China | + | + | + |
| *E. faecalis* 20170714-1 | Unknow | + | + | + |
| *E. faecalis* 20170714-2 | Unknow | + | + | + |
| *E. faecalis* 20170714-3 | Unknow | - | - | - |
| *E. faecium* HBM101 | Hubei, China | - | - | - |
| *E. faecium* HBM1-2 | Hubei, China | - | - | - |
| *E. faecium* 14409 | Beijing, China | - | - | - |
| *E. faecium* STT-03 | Hainan, China | - | - | - |
| *E. faecium* STT-04 | Hainan, China | - | - | - |
| *E. faecium* 21905 | Hubei, China | - | - | - |
| *E. faecium* 180724 | Unknow | - | - | - |
| *E. faecium* 180801 | Unknow | - | - | - |
| *E. faecium* SX11 | Shanxi, China | - | - | - |
| *E. faecium* HN12 | Hainan, China | - | - | - |
| *E. coli* 18701 | Hubei, China | - | - | - |
| *E. coli* 14997 | Hubei, China | - | - | - |
| *E. coli* O157:H7 | Hubei, China | - | - | - |
| *E. coli* DH5α | Hubei, China | - | - | - |
| *Salmonella* 268 | Hubei, China | - | - | - |
| *Salmonella* 140411 | Hubei, China | - | - | - |
| *Salmonella* 1003 | Hubei, China | - | - | - |

NOTE:(**+**) indicates that plaques were observed ;(**-**) indicates no plaques were observed.

**Table S2 Sequence information for the *E. faecalis* phages belonging to Saphexavirus subfamily used in this study.**

| Name | GenBank | Length | Identity | G+C% | ORF | tRNA | Countries |
|---|---|---|---|---|---|---|---|
| *Enterococcus* phage vB_EfaS_PHB08 | MK570225.1 | 55,244 bp | 100% | 40.00% | 91 | 1 | China |
| *Enterococcus* phage vB_EfaS_IME198 | KT932699.1 | 58,000 bp | 96.18% | 40.02% | 95 | 0 | China |
| *Enterococcus* phage vB_EfaS_HEf13 | MH618488.1 | 57,811 bp | 95.95% | 40.03% | 95 | 1 | South Korea |
| *Enterococcus* phage vB_EfaS_Ef7.1 | MK721194.1 | 58,018 bp | 94.09% | 40.03% | 102 | 3 | The United States |
| *Enterococcus* phage EF-P29 | KY303907.1 | 58,984 bp | 96.07% | 39.77% | 101 | 0 | China |
| *Enterococcus* phage EF-P10 | KY472224.1 | 57,408 bp | 96.05% | 39.82% | 127 | 0 | China |
| *Enterococcus* phage VD13 | KJ127303.1 | 55,726 bp | 94.89% | 40.01% | 88 | 1 | The United States |
| *Enterococcus* phage IME-EF1 | NC_041959 | 57,081 bp | 96.40% | 40.05% | 98 | 0 | The United States |
| *Enterococcus* phage SAP6 | NC_041960 | 58,619 bp | 97.53% | 40.00% | 44 | 0 | South Korea |
| *Enterococcus* phage vB_EfaS_EF1c55 | MN103542.1 | 55,876 bp | 89.20% | 39.79% | 94 | 0 | Poland |
| *Enterococcus* phage BC-611 | AB712291.1 | 53,996 bp | 95.40% | 40.45% | 88 | 1 | Japan |
| *Enterococcus* phage Entf1 | MK800154.1 | 58,938 bp | 95.24% | 39.93% | 105 | 1 | Russia |
| *Enterococcus* phage vB_EfaS_Ef2.2 | MK721189.1 | 58,400 bp | 89.52% | 39.92% | 103 | 2 | The United States |

**Table S3 Gene annotation for each CDS of PHB08.**

| CDS | Start | Stop | Length (bp) | Size (aa) | Function | Accession numbers | % identity | *E* value |
|---|---|---|---|---|---|---|---|---|
| 1 | 151 | 17 | 135 | 44 | hypothetical protein | -- | -- | -- |
| 2 | 603 | 229 | 375 | 124 | hypothetical protein | -- | -- | -- |
| 3 | 982 | 596 | 387 | 128 | hypothetical protein | -- | -- | -- |
| 4 | 1406 | 1083 | 324 | 107 | hypothetical protein | -- | -- | -- |
| 5 | 2683 | 2171 | 513 | 170 | hypothetical protein | -- | -- | -- |
| 6 | 2991 | 2686 | 306 | 101 | Phage DNA binding protein | YP_009603915.1 | 99.01% | 2e-71 |
| 7 | 3259 | 2993 | 267 | 88 | hypothetical protein | -- | -- | -- |
| 8 | 3479 | 3252 | 228 | 75 | hypothetical protein | -- | -- | -- |
| 9 | 3722 | 3513 | 210 | 69 | hypothetical protein | -- | -- | -- |
| 10 | 3947 | 3735 | 213 | 70 | hypothetical protein | -- | -- | -- |
| 11 | 4207 | 3947 | 261 | 86 | hypothetical protein | -- | -- | -- |
| 12 | 4398 | 4204 | 195 | 64 | hypothetical protein | -- | -- | -- |
| 13 | 4895 | 4476 | 420 | 139 | hypothetical protein | -- | -- | -- |
| 14 | 5109 | 4909 | 201 | 66 | hypothetical protein | -- | -- | -- |
| 15 | 5707 | 5120 | 588 | 195 | hypothetical protein | -- | -- | -- |
| 16 | 6053 | 5730 | 324 | 107 | hypothetical protein | -- | -- | -- |
| 17 | 6259 | 6023 | 237 | 78 | hypothetical protein | -- | -- | -- |
| 18 | 6521 | 6243 | 279 | 92 | hypothetical protein | -- | -- | -- |
| 19 | 7275 | 6580 | 696 | 231 | hypothetical protein | -- | -- | -- |
| 20 | 7663 | 7268 | 396 | 131 | hypothetical protein | -- | -- | -- |
| 21 | 8120 | 7665 | 456 | 151 | hypothetical protein | -- | -- | -- |
| 22 | 10726 | 8195 | 2532 | 843 | DNA polymerase I | YP_009218931.1 | 98.34% | 0.0 |
| 23 | 10991 | 10806 | 186 | 61 | hypothetical protein | -- | -- | -- |
| 24 | 11232 | 11005 | 228 | 75 | hypothetical protein | -- | -- | -- |
| 25 | 11585 | 11232 | 354 | 117 | hypothetical protein | -- | -- | -- |
| 26 | 11798 | 11586 | 213 | 70 | hypothetical protein | -- | -- | -- |
| 27 | 12010 | 11801 | 210 | 69 | hypothetical protein | -- | -- | -- |
| 28 | 12231 | 12007 | 225 | 74 | hypothetical protein | -- | -- | -- |
| 29 | 12439 | 12245 | 195 | 64 | hypothetical protein | -- | -- | -- |
| 30 | 12620 | 12441 | 180 | 59 | hypothetical protein | -- | -- | -- |
| 31 | 13026 | 12820 | 207 | 68 | hypothetical protein | -- | -- | -- |
| 32 | 13154 | 13038 | 117 | 38 | hypothetical protein | -- | -- | -- |
| 33 | 13720 | 13157 | 564 | 187 | hypothetical protein | -- | -- | -- |
| 34 | 14444 | 13812 | 633 | 210 | hypothetical protein | -- | -- | -- |
| 35 | 15006 | 14437 | 570 | 189 | adenylate kinase and related kinases | -- | -- | -- |
| 36 | 15437 | 15003 | 435 | 144 | hypothetical protein | -- | -- | -- |
| 37 | 15903 | 15574 | 330 | 109 | hypothetical protein | -- | -- | -- |
| 38 | 16931 | 15903 | 1029 | 342 | hypothetical protein | -- | -- | -- |
| 39 | 17364 | 16924 | 441 | 146 | HNH homing endonuclease | NP_389885.1 | 28.46% | 1e-05 |
| 40 | 17661 | 17437 | 225 | 74 | hypothetical protein | | | |
| 41 | 19040 | 17676 | 1365 | 454 | Replicative DNA helicase | NP_719448.1 | 24.37% | 8e-04 |

| | | | | | | | | |
|---|---|---|---|---|---|---|---|---|
| | | | | | (DnaB) | | | |
| 42 | 19828 | 19052 | 777 | 258 | DNA replication protein | YP_009036407.1 | 99.61% | 0.0 |
| 43 | 20230 | 19877 | 354 | 117 | hypothetical protein | -- | -- | -- |
| 44 | 21252 | 20305 | 948 | 315 | DNA primase | -- | -- | -- |
| 45 | 21452 | 21264 | 189 | 62 | hypothetical protein | -- | -- | -- |
| 46 | 21610 | 21452 | 159 | 52 | hypothetical protein | -- | -- | -- |
| 47 | 21861 | 21607 | 255 | 84 | hypothetical protein | -- | -- | -- |
| 48 | 22253 | 21861 | 393 | 130 | hypothetical protein | -- | -- | -- |
| 49 | 22401 | 22255 | 147 | 48 | hypothetical protein | -- | -- | -- |
| 50 | 22585 | 22373 | 213 | 70 | hypothetical protein | -- | -- | -- |
| 51 | 23471 | 23346 | 126 | 41 | hypothetical protein | -- | -- | -- |
| 52 | 23856 | 23656 | 201 | 66 | hypothetical protein | -- | -- | -- |
| 53 | 24242 | 23856 | 387 | 128 | hypothetical protein | -- | -- | -- |
| 54 | 24679 | 24245 | 435 | 144 | hypothetical protein | -- | -- | -- |
| 55 | 25556 | 24732 | 825 | 274 | hypothetical protein | -- | -- | -- |
| 56 | 26060 | 26284 | 225 | 74 | hypothetical protein | -- | -- | -- |
| 57 | 26286 | 26714 | 429 | 142 | hypothetical protein | -- | -- | -- |
| 58 | 26732 | 27292 | 561 | 186 | HNH homing endonuclease | QDB70581.1 | 97.85% | 2e-138 |
| 59 | 28035 | 27319 | 717 | 238 | Phage lysin | -- | -- | -- |
| 60 | 31337 | 28110 | 3228 | 1075 | Phage minor structural protein | QBZ69423.1 | 76.49% | 0.0 |
| 61 | 35341 | 31349 | 3993 | 1330 | Phage tail fibers | YP_009604020.1 | 87.17% | 0.0 |
| 62 | 38240 | 35355 | 2886 | 961 | Phage tail length tape-measure protein | YP_009218894.1 | 98.96% | 0.0 |
| 63 | 38477 | 38253 | 225 | 74 | hypothetical protein | -- | -- | -- |
| 64 | 38928 | 38488 | 441 | 146 | hypothetical protein | -- | -- | -- |
| 65 | 39761 | 39072 | 690 | 229 | hypothetical protein | -- | -- | -- |
| 66 | 40216 | 39782 | 435 | 144 | hypothetical protein | -- | -- | -- |
| 67 | 40609 | 40229 | 381 | 126 | hypothetical protein | -- | -- | -- |
| 68 | 40971 | 40594 | 378 | 125 | hypothetical protein | -- | -- | -- |
| 69 | 41391 | 40987 | 405 | 134 | hypothetical protein | -- | -- | -- |
| 70 | 41891 | 41451 | 441 | 146 | Chitinase (EC 3.2.1.14) | AYH92720.1 | 97.95% | 2e-100 |
| 71 | 42852 | 42046 | 807 | 268 | Phage major capsid protein | YP_006488741.1 | 99.63% | 0.0 |
| 72 | 43575 | 42901 | 675 | 224 | hypothetical protein | -- | -- | -- |
| 73 | 44441 | 43686 | 756 | 251 | hypothetical protein | -- | -- | -- |
| 74 | 45988 | 44453 | 1536 | 511 | Phage portal protein | AYH92724.1 | 99.80% | 0.0 |
| 75 | 47316 | 46045 | 1272 | 423 | Phage terminase, large subunit | YP_009603889.1 | 99.53% | 0.0 |
| 76 | 47627 | 47379 | 249 | 82 | hypothetical protein | -- | -- | -- |
| 77 | 47990 | 47646 | 345 | 114 | hypothetical protein | -- | -- | -- |
| 78 | 48603 | 48004 | 600 | 199 | hypothetical protein | -- | -- | -- |
| 79 | 48884 | 49198 | 315 | 104 | hypothetical protein | -- | -- | -- |
| 80 | 49198 | 49455 | 258 | 85 | hypothetical protein | -- | -- | -- |
| 81 | 49455 | 49847 | 393 | 131 | hypothetical protein | -- | -- | -- |

| | | | | | | | | |
|---|---|---|---|---|---|---|---|---|
| 82 | 49849 | 50235 | 387 | 128 | hypothetical protein | -- | -- | -- |
| 83 | 50232 | 50492 | 261 | 86 | hypothetical protein | -- | -- | -- |
| 84 | 50494 | 50700 | 207 | 68 | hypothetical protein | -- | -- | -- |
| 85 | 50690 | 50992 | 303 | 100 | hypothetical protein | -- | -- | -- |
| 86 | 51012 | 51380 | 369 | 121 | hypothetical protein | -- | -- | -- |
| 87 | 51824 | 52582 | 759 | 252 | HNH homing endonuclease | APU00279.1 | 98.81% | 0.0 |
| 88 | 52657 | 53511 | 855 | 284 | hypothetical protein | -- | -- | -- |
| 89 | 53512 | 53730 | 219 | 72 | hypothetical protein | -- | -- | -- |
| 90 | 53732 | 53944 | 213 | 70 | hypothetical protein | -- | -- | -- |
| 91 | 54122 | 54262 | 141 | 46 | hypothetical protein | -- | -- | -- |
